# Investigation of Viscoelastic Properties of Macrophage Membrane–Cytoskeleton Induced by Gold Nanorods in Leishmania Infection

**DOI:** 10.3390/nano15171373

**Published:** 2025-09-05

**Authors:** Maria L. B. Pertence, Marina V. Guedes, Rosimeire C. Barcelos, Jeronimo N. Rugani, Rodrigo P. Soares, Joyce L. V. Cruz, Alessandra M. de Sousa, Rubens L. do Monte-Neto, Livia G. Siman, Anna C. P. Lage, Ubirajara Agero

**Affiliations:** 1 Department of Physics, Federal University of Minas Gerais, Av. Antônio Carlos 6627, Pampulha, Belo Horizonte 31270-901, MG, Brazil; mpertence@fis.dout.ufmg.br (M.L.B.P.); marinavguedes@ufmg.br (M.V.G.); liviasg@fisica.ufmg.br (L.G.S.); 2 Department of Chemistry, Federal University of São João del-Rei, Praça Dom Helvécio 74, Centro, São João del-Rei 36301-160, MG, Brazil; rosicbarcelos@ufsj.edu.br; 3 René Rachou Institute, Oswaldo Cruz Foundation, Av. Augusto de Lima 1715, Barro Preto, Belo Horizonte 30190-002, MG, Brazil; jeronimomnr@hotmail.com (J.N.R.); rodrigo.pedro@fiocruz.br (R.P.S.); joycevianac@gmail.com (J.L.V.C.); alessandra.sousa@fiocruz.br (A.M.d.S.); rubens.monte@fiocruz.br (R.L.d.M.-N.)

**Keywords:** gold nanorods, defocusing microscopy, viscoelastic properties, leishmaniasis, quantitative phase microscopy, cell biomechanics

## Abstract

Cell membranes and the cytoskeleton play crucial roles in the regulation of cellular responses by mediating mechanical forces and physical stimuli from the microenvironment through their viscoelastic properties. Investigating these properties provides valuable insights into disease mechanisms and therapeutic strategies. Gold nanorods (GNRs), especially under irradiation, exhibit lethal effects against *Leishmania* parasites through plasmonic photothermal conversion. In this study, we focus on evaluating the effects of non-irradiated GNRs on macrophage properties to better understand their intrinsic interactions with cells and support the development of future phototherapy applications. Here, defocusing microscopy (DM), a quantitative phase microscopy technique, was used to analyze membrane fluctuations in macrophages (M∅s) exposed to GNRs (average length of 43±8 nm and diameter of 20±4 nm) and infected with *Leishmania amazonensis*. By quantifying membrane–cytoskeleton fluctuation from defocused images, we extracted viscoelastic parameters, including bending modulus ($k_{c}$) and viscosity (η), to characterize membrane behavior in detail. Our results show that infection increases both $k_{c}$ and η, while treatment at IC_50_ reduces infection and selectively increases $k_{c}$ without affecting η. In healthy macrophages, exposure to GNRs resulted in a reduction in both parameters, indicative of increased membrane fluidity and cytoskeletal rearrangement. These findings provide new insights into the biomechanical effects of GNRs on macrophages and may enlighten the design of future phototherapeutic approaches.

## 1. Introduction

Leishmaniasis is a group of diseases transmitted by infected female phlebotomine sandflies and is recognized as a serious emerging and neglected global health problem [1]. Its most common form, cutaneous leishmaniasis (CL), is endemic in more than 98 countries, with approximately 12 million cases and 2.5 million new cases reported annually [1].

Current treatments for CL are largely limited to expensive drugs that often cause adverse effects, a situation worsened by the emergence of drug-resistant parasite strains, which compromise efficacy and patient adherence [2,3,4]. Treatment options vary according to geographic location, *Leishmania* species, and clinical presentation. The most commonly used systemic therapies include pentavalent antimonials, such as sodium stibogluconate and meglumine antimoniate, considered first-line in many regions, especially in developing countries. In severe or resistant cases, amphotericin B formulations are used, while miltefosine, an oral drug, has shown efficacy, particularly in cases in New World [5,6,7].

Gold nanorods (GNRs) have emerged as promising nanosystems due to their intrinsic antiparasitic activity against *Leishmania* species [2,8]. As GNRs alone do not completely eradicate the infection, their efficacy is further enhanced when combined with irradiation, exploiting their unique optical properties derived from surface plasmon resonance (SPR) [9]. Upon irradiation, GNRs convert near-infrared light into localized heat through rapid photothermal effects, effectively damaging parasites and infected cells [9,10], particularly in cutaneous leishmaniasis applications where light penetration is minimally limited.

Beyond these photothermal applications, the biological effects of GNRs in the absence of irradiation warrant careful investigation. Their interaction with cells can induce cytotoxic responses influenced by parameters such as concentration, surface chemistry, and cell type. Although their internalization through mechanisms like endocytosis and pinocytosis is well documented, the specific impact of GNRs alone on cellular biomechanics, both during and after uptake, remains insufficiently explored [11,12].

In the study of biological systems, the analysis of changes in intracellular components, such as the plasma membrane and attached cytoskeleton, is essential to understand the interactions of drugs or pathogen with cells. In addition to playing a crucial role in the mechanical properties of cells and tissues organization [13], these structures enable cells to respond to microenvironmental stimuli, including tension, shear stress, hydrostatic pressure, and compression, through physical processes described by the viscoelastic properties of cell dynamics [14,15].

Investigating variations in these properties, particularly in macrophages (M∅s), immune cells derived from the bone marrow [13], is fundamental to understanding disease progression, elucidating cellular processes, and guiding the development of novel therapeutic strategies [8,16]. Evaluating how GNRs interact with cells without irradiation provides valuable insights into their intrinsic effects on cellular biomechanics and establishes a basis for future research into photothermal applications.

In this context, traditional methods used to investigate cellular mechanics, such as atomic force microscopy (AFM) and optical tweezers, although precise, rely on direct mechanical manipulation [17,18,19]. Fluorescence-based approaches often require labeling agents that can alter cell dynamics or induce phototoxicity [20,21,22,23]. In contrast, phase imaging techniques offer powerful tools for real-time analysis of membrane dynamics [24], such as defocusing microscopy (DM), a non-invasive and label-free method that preserves the native physiological conditions of the sample [25,26].

Most biological materials, including cells, are considered phase objects, because they do not absorb a significant amount of light when observed under an optical microscope. In other words, when in focus, these samples do not exhibit discernible variations in light intensity [25,26]. In DM, the observed contrast of phase objects is zero in the focal plane and becomes visible when the sample is defocused, reflecting local variations in the fluctuations in the membrane–cytoskeleton associated with the viscoelastic properties of the cell. These fluctuations allow for quantification of parameters, such as the bending modulus ($k_{c}$), indicating the cell’s surface resistance to curvature, and viscosity (η), related to the resistance of the fluid to flow [27,28].

In this study, DM was used to explore the mechanical behavior of macrophages exposed to gold nanorods and infected with *Leishmania amazonensis*, a causative agent of CL. This approach aimed to advance the understanding of the interactions between cells and nanoparticles relevant to therapeutic strategies. The findings contribute to a broader understanding of *Leishmania*–cell interactions by assessing changes in cell viscoelasticity and evaluating the intrinsic effects of GNRs, potentially informing future research on photothermal applications.

## 2. Materials and Methods

### 2.1. Cell Lines and Parasite Strains

The THP-1 macrophage cell line (American Type Culture Collection ATCC^®^ TIB-202™) [29] was provided by the René Rachou Research Institute. In this study, the reference strain of the World Health Organization (WHO), *Leishmania (Leishmania) amazonensis* (IFLA/BR/1967/PH8) was used. These parasites were previously transfected with constructs pIR1SAT-LUC(a)-DsRed2(b) (B5947) [30], which contain the red fluorescent protein (RFP) gene DsRed2, encoding a protein that emits red fluorescence at a wavelength of 590 nm when excited by green light at 546 nm. The strain was typed as previously described [31] and was also provided by the René Rachou Research Institute.

### 2.2. Chemical Reagents and Solutions

Gold nanorods were synthesized using reagents acquired from Sigma-Aldrich (St. Louis, MO, USA): chloroauric acid (HAuCl_4_, ≥99.0%), cetyltrimethylammonium bromide (CTAB, ≥98.0%), silver nitrate (AgNO_3_, ≥99.0%), and sodium borohydride (NaBH_4_, ≥96.0%). Resveratrol (≥98%) was obtained as a pharmaceutical-grade compound imported from China by Fagron B.R. (São Paulo, Brazil). Analytical-grade ethanol was used without further purification. All solutions were prepared with ultrapure Milli-Q water (resistivity ρ = 18 MΩ·cm) produced by a Millipore system (Milipore, Burlington, MA, USA).

### 2.3. Cell Culture and Parasite Maintenance Protocols

THP-1 macrophages were cultured in RPMI 1640 medium (Sigma, St. Louis, MO, USA) supplemented with hypoxanthine, HEPES, dextrose, sodium bicarbonate, L-glutamine, and 10% fetal bovine serum. Cultures were maintained at 37 °C, 95% humidity, and 5% CO_2_. Cell viability and count were routinely assessed using the trypan blue exclusion method.

To maintain infectivity, *Leishmania amazonensis* parasites were continuously passaged in BALB/c mice (*Mus musculus*), and amastigotes were isolated from footpad lesions [32]. Subsequently, these were differentiated into promastigotes, cultured in T25 cell culture flasks containing alpha-MEM medium (Sigma) supplemented with 10% fetal bovine serum, 5 µg/mL hemin, and 5 µM biopterin, at pH 7 and 25 °C. Parasite passages were performed every three days, using 1 × 10^6^ parasites per inoculum in 5 mL of complete alpha-MEM medium. For long-term storage, the parasites were cryopreserved in a medium containing 20% fetal bovine serum and 10% dimethyl sulfoxide (DMSO) at −80 °C [31,32].

For differentiation, 4 × 10^5^ THP-1 cells (2 × 10^5^ cells/mL in 2 mL per well) were seeded in 35 mm glass bottom dishes (Cellvis) with a 20 mm diameter coverslip at the bottom and incubated for 72 h in RPMI 1640 medium supplemented with 50 ng/mL phorbol 12-myristate 13-acetate (PMA) [33].

THP-1 macrophages were infected with *Leishmania* promastigotes in a 10:1 parasite-to-cell ratio for 4 h [34]. Following infection, the cells were washed with RPMI medium to remove extracellular parasites. The infected macrophages were then treated with a concentration equivalent to the IC_50_, adjusted for CTAB content, and incubated under the same culture conditions for subsequent analysis by defocusing and fluorescence microscopy.

Our research group previously established cytotoxicity and anti-amastigote assays using validated protocols with differentiated THP-1 cells and *Leishmania* infection models. CC_50_ and IC_50_ values were determined on the basis of the results obtained in these assays [8]. The IC_50_ concentration, defined as the concentration required to inhibit 50% of parasite proliferation, was established as 2.8 μM of Au, equivalent to 4.2 pM of GNRs. The CC_50_ concentration, defined as the concentration that reduces 50% of THP-1 cell viability, was 280 μM of Au, equivalent to 42 pM of GNRs. The ratio between CC_50_ and IC_50_ values, known as the selectivity index (SI), is a parameter used to evaluate the safety and efficacy of compounds. For this study, the SI was greater than 100, which is highly relevant from both a biological and clinical perspective, as a selectivity index ≥10 is considered desirable for leishmanicidal compounds. This indicates that GNRs exhibit strong antiparasitic activity with minimal cytotoxicity to host cells [8,35].

### 2.4. Gold Nanorod Synthesis and Physicochemical Characterization

GNRs were synthesized according to a seedless synthesis protocol adapted from [36,37]. The growth solution was prepared by gradual mixing of the aqueous CTAB solution, the aqueous HAuCl_4_ solution, the aqueous AgNO_3_ solution and alcoholic resveratrol solution at 25–30 °C. The growth of GNRs was triggered by the injection of a freshly prepared cold aqueous NaBH_4_ solution. The resulting solution was kept intact in a 70 °C water bath for 4 h to produce GNRs. CTAB, HAuCl_4_, AgNO_3_, resveratrol, and NaBH_4_ concentrations in the final mixture were 50, 0.5, 0.06, 5, and 0.012 mM, respectively.

Due to the fact that CTAB is toxic to biological materials in large quantities, GNRs need to undergo a calibration process, which is necessary to reduce the cytotoxicity. This was performed following the injection water resuspension protocol described in [37], which aims to eliminate residual CTAB as much as possible while preserving the morphological stability of the GNRs and maintaining cell viability.

GNRs were characterized by UV-VIS spectroscopy using a Varioskan™ LUX multimode reader (Thermo Fisher Scientific, Waltham, MA, USA). Transmission electron microscopy (TEM) images were obtained using a Tecnai G2-20 SuperTwin electron microscope (FEI, Hillsbora, OR, USA) operated at 200 kV. The zeta potential (ζ-potential) of all samples was analyzed using a Litesizer™ 500 (Anton Paar GmbH, Graz, Austria) with an electrophoretic mobility cell.

### 2.5. Microscopy Equipment and Imaging Setup

Cells were analyzed using defocusing microscopy on an inverted microscope (Nikon Eclipse Ti-ϵ). The microscope was equipped (see Figure S1 in Supplementary Materials) with an incubation system (LCI—live cell instrument, air–gas mixer, and CU-109 controller) that provided a stable environment for the cells with 37 °C, 95% humidity, and 5% CO_2_. These features ensured that the samples remained under controlled environmental conditions throughout the measurements.

The images were acquired using an oil immersion objective (Nikon CFI Apochromat TIRF 100× Oil; Nikon Corporation, Tokyo, Japan) with 100× magnification and a numerical aperture (NA) of 1.49, as well as a 10× objective (Nikon CFI Achromat ADL 10×) with an NA of 0.25 for observation of wide-field samples. For epifluorescence imaging, the same objectives were used in fluorescence microscopy, combined with a mercury lamp (Nikon Intensilight C-HGFI; Nikon Corporation, Tokyo, Japan) and a set of G-2E/C filters (bandpass emission).

Images and videos were captured using a CCD camera (Uniq UP1800 CL, 12-bit; Uniq Vision, Santa Clara, CA, USA). To ensure focus stability throughout the experiments, the Nikon Perfect Focus System (PFS) was used. All videos were recorded using the 100× objective, for which the pixel size of the Uniq camera corresponds to 0.064 µm × 0.064 µm.

### 2.6. Defocusing Microscopy

The theoretical framework of defocusing microscopy used in this study incorporates elements of Fourier optics, Fresnel diffraction, and previous analytical formulations developed for this method [25,38,39,40]. In this technique, the observed image contrast results from the propagation and diffraction of the electric field through a phase object captured at defocused planes in the optical path of the microscope.

To quantify membrane mechanics, we employed a dynamic mathematical model, which describes contrast fluctuations through contrast temporal autocorrelation functions (CTAF) in selected regions of interest. These fluctuations were analyzed to extract the parameters. The fitting model was developed using an approximate analytical expression for the contrast of a defocused cell from [25], adapted for our experimental conditions. The original equation in [25] describes the contrast of a cell, considering two interfaces, the upper and lower membranes. In our model, since one membrane adheres to the substrate, we consider only the fluctuations of the upper membrane, which is free to undergo thermal motion. Based on this assumption, the contrast $C\left(\vec{\rho }\right)$ can be expressed as

(1) $$\;\;\;\;C\left(\vec{\rho }\right)=\frac{{\left|E\left(\vec{\rho }\right)\right|}^{2}-{E_{0}}^{2}}{{\left|E_{0}\right|}^{2}}\approx \frac{2∆nk_{0}}{\sqrt{S}}\sum_{\vec{q}}\left[h\left(\vec{q}\right)sen\left(\vec{q}\cdot \vec{\rho }\right)sen\left(\frac{z_{f}}{2k}q^{2}\right)\right]\;$$

**Figure 1:**
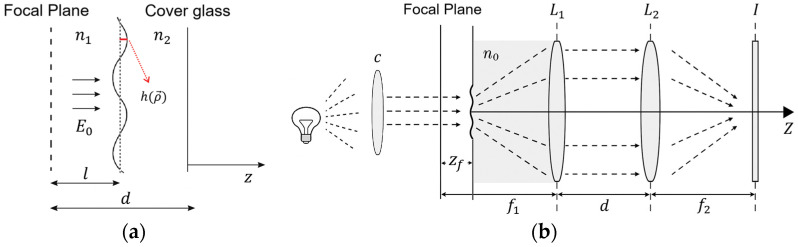
Schematic representation of the optical geometry in defocusing microscopy. (**a**) Geometry of the propagation of the electric field $E_{0}$ through the phase object, showing the interface ripple profile $h\left(\vec{\rho }\right)$, the refractive indices $n_{1}$ and $n_{2}$, and the cover glass. L and *d* denote characteristic distances, and the focal plane is indicated. (**b**) Optical path in the defocused microscope. Caption: C: condenser; $L_{1}$: objective; $L_{2}$: tube lens. $n_{0}$: refractive index of the immersion medium; $z_{f}$: defocus amount; $f_{1},f_{2}:$ focal lengths; *d*: distance between lenses and I: image plane.

In this equation, ρ represents the position vector and $\vec{q}$ is the wave vector. The difference in the refractive index between cells, considered phase objects, and the surrounding cell culture medium is given by ∆n, while n  corresponds to the refractive index of the medium. The parameters $k_{0}$ and k correspond to the wave number of light in vacuum and in the medium, respectively. The ripple profile of the phase object is described either by $h\left(\vec{\rho }\right)$ in real space or by $h\left(\vec{q}\right)$ in the corresponding wave space, and the surface of the interface is indicated by S. The term $z_{f}$ defines the distance at which the phase object is defocused in the microscope, allowing for sample visualization. $E\left(\vec{\rho }\right)$ denotes the electric field transmitted by the cell, and $E_{0}$ denotes the undiffracted electric field. Figure 1 illustrates the structures and parameters described in Equation (1).

The development of Equation (1), fully detailed in Text S1 in Supplementary Materials, leads to the determination of the CTAF associated with viscoelastic parameters:

(2) $$<∆C\left(0,0\right)∆C\left(0,t\right)>={\left(\frac{∆n}{n_{0}}\right)}^{2}\left(\frac{z_{f}^{2}}{2\pi }\right)\left(\frac{k_{b}T}{k_{c}}\right)\int_{0}^{q_{max}}{qe}^{\frac{-k_{c}q^{3}t}{4\eta }}dq$$

In this expression, the refractive index of the immersion oil used in the objective was measured as $n_{0}=1508\pm 0.0003$, and the difference in the refractive index between the cell and the medium was taken as ∆n=0.06, with $k_{b}T=4\times 10^{-21}\;J$. The amount of defocus was standardized at $z_{f}=1\;\mu m$. To fit the experimental data, we subtracted a constant, which corresponds to an experimentally observed background fluctuation of the order of $\sim 10^{-5}$, and may arise from intracellular structures with varying contrast moving in the analysis region.

For our optical microscope setup, the upper limit of the wave number ($q_{max})$ was determined through computational tests using red blood cells as a reference object. By analyzing the point spread function and estimating the diameter of the Airy disk, we established a practical resolution limit corresponding to $q_{max}\sim 4\;{\mu m}^{-1}$. This value was adopted in our analysis as the upper bound of the accessible spatial frequency range, as it accurately reflects the true resolving power of the microscope.

DM measurements were conducted within the first 5 h of the 24 h incubation period with gold nanorods to characterize membrane fluctuations between the different experimental groups: healthy, infected, healthy incubated with GNRs, and infected incubated with GNRs. Cell videos were recorded to monitor membrane fluctuations, each lasting 10 min, with a capture rate of 2 frames per second. Recordings were performed every 15 min throughout the analysis period.

The videos were analyzed using the publicly available ImageJ 1.54d software [41], with a custom plugin developed by the Mesquita group [26]. For each video, at least three regions of the adhered portion of the cell membrane were selected to extract autocorrelation curves for analysis, carefully excluding internal cellular structures, such as lysosomes and other organelles, as well as particles exhibiting Brownian motion or residual *Leishmania* parasites from the infection process.

### 2.7. Fluorescence Microscopy

Fluorescence microscopy analysis was performed to monitor the progression of infection and assess the impact of the gold nanorods. Measurements were carried out in triplicate at the beginning of treatment, and subsequently at 5, 24, 48, and 72 h. To ensure accuracy, samples were washed every 24 h to remove non-internalized parasites and dead cells. Consequently, GNRs in solution were removed after 24 h of incubation.

The corrected total cell fluorescence (CTCF) was used to quantify the fluorescence signal attributed to the parasites, effectively eliminating background interference. This quantification was performed using ImageJ software, according to the method described in [42], using the following equation:

(3) $$CTCF=\sum \left(IntDen-\left(A\times Mean\;fluorescence\;of\;background\right)\right)\;$$

where Integrated Density (*IntDen*) represents the sum of pixel intensity values within the fluorescence area, A is the area of the fluorescence region (in pixels^2^), and *Mean fluorescence of background* refers to the average fluorescence intensity measured in a cell-free region, used to estimate background noise.

For each sample, measurements were performed in triplicate and reported as mean ± standard deviation (see Table S1 in Supplementary Materials). The CTCF values obtained from ImageJ, expressed in arbitrary units (a.u.). Data were collected for each time point and experimental conditions and graphical representations were generated using Python (version 3.11.9).

### 2.8. Data Processing and Statistical Analysis

The CTAF curves obtained from the DM data were fitted using Equation (2) with MATLAB R2024a (The MathWorks, Natick, MA, USA) software. From this fit, we obtain the parameters $k_{c}$ and η, which were used to characterize the membrane–cytoskeleton fluctuations between all experimental groups (see Section 2.7). The data fitting procedure involved non-linear curve fitting, and the quality of the fits was evaluated using R^2^ values and residual analysis.

To validate and interpret the results obtained from defocused microscopy (MATLAB analysis), statistical analyses were performed. For each group, the mean, standard deviation, median, and interquartile range (IQR) were calculated. Normality was assessed using the Shapiro–Wilk test. A *p*-value < 0.05 was considered statistically significant. Given the non-normal distribution of the data, the Mann–Whitney U test, a non-parametric method to compare independent groups, was employed. These statistical analyses were performed using Python and are presented and discussed in the following section.

## 3. Results

### 3.1. GNRs Syntheses and Physicochemical Characterizations

**Figure 2:**
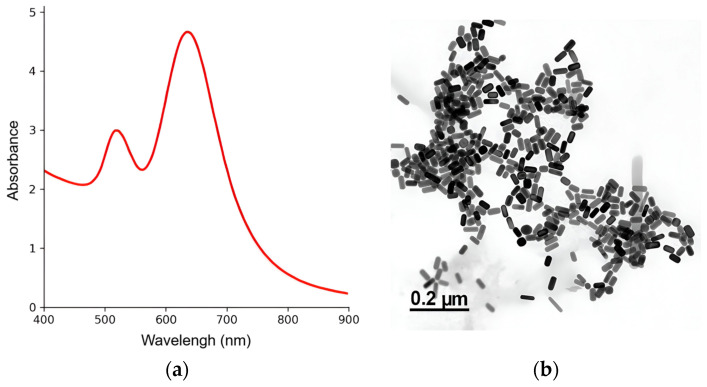
Characterization of GNRs through spectroscopy and TEM techniques. (**a**) UV–Vis absorption spectrum displaying the characteristic transverse and longitudinal plasmon resonance bands of the GNRs. (**b**) TEM image showing the morphology and size distribution of the nanorods.

The synthesis of GNRs was confirmed using a combination of complementary techniques. UV–Vis absorption spectroscopy revealed a transverse plasmon resonance band at 520 nm and a longitudinal band at 636 nm, both characteristic of GNRs (Figure 2a).

Transmission electron microscopy analysis of 161 particles confirmed the successful formation of uniform rod-shaped structures, with an average aspect ratio (AR) of approximately 2.0 and a narrow size distribution. A small fraction of amorphous structures, resulting from the synthesis process, was also observed, but was negligible and did not interfere with the application (Figure 2b). Further physicochemical characterization indicated that the GNRs had an average length of 43±8 nm and an average diameter of 20±4 nm.

The measured zeta potential (ζ-potential) was 55.1±0.8 mV, suggesting good colloidal stability under the tested conditions and reducing the risk of aggregation during subsequent cellular applications or biological assays. Both the aspect ratio and size distribution agree with previously reported data, supporting the reproducibility and reliability of the synthesis protocol employed.

### 3.2. Fluorescence Microscopy Analysis

**Figure 3:**
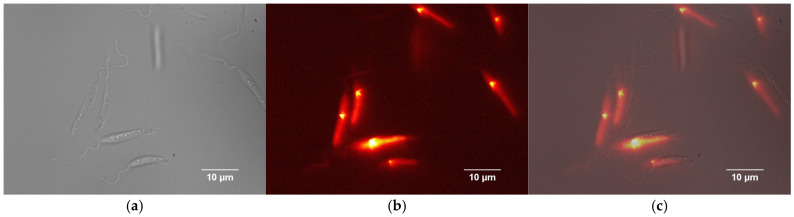
Representative images of *Leishmania (Leishmania) amazonensis* in their promastigote form expressing the red fluorescent protein DsRed2. (**a**) Bright-field image showing the parasite morphology. (**b**) Fluorescence microscopy image of the same parasite displaying red fluorescence emission. (**c**) Merged image combining bright-field and fluorescence channels, illustrating the spatial correlation between parasite morphology and fluorescence signal.

In our study, fluorescence microscopy was essential to monitor the progression of *Leishmania amazonensis* infection and to evaluate the impact on host cells over time, including under treatment conditions. At the early stage of infection, the parasites are in their extracellular promastigote form, which is easily visualized in bright-field microscopy due to their characteristic elongated morphology (Figure 3a). These promastigotes express the red fluorescent protein DsRed2, allowing fluorescence detection (Figure 3b). However, once internalized by host cells and differentiated into amastigotes, the parasites became less distinguishable in bright-field images.

**Figure 4:**
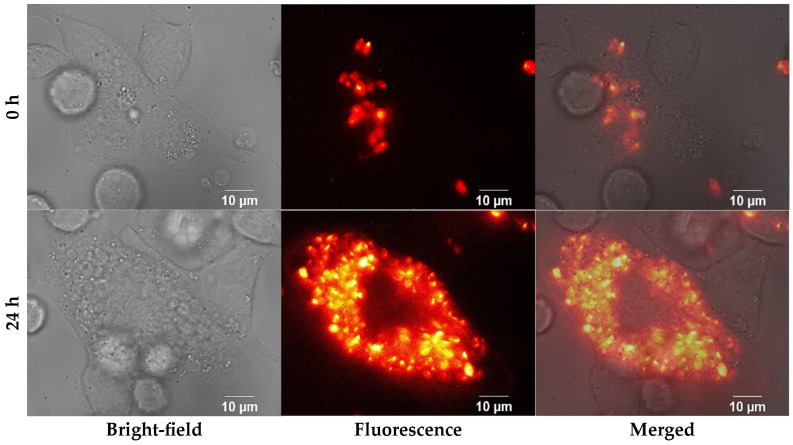
Bright-field, fluorescence, and merged microscopy images showing the progression of infection in THP-1 macrophages. The top row presents images from the onset of infection, with bright-field images on the left, fluorescence images in the center, and the merged (overlay) images in the right column illustrating colocalization. The bottom row shows the corresponding images 24 h after infection, arranged in the same order (left—bright-field, center—fluorescence, right—merged). The merged images demonstrate the spatial overlap of the infection markers with the cell morphology.

In this context, fluorescence microscopy was essential to detect and track intracellular parasites during the initial stages of infection (Figure 4). Initially, parasites could only be identified by their fluorescence signal. As the infection progressed, fluorescence images revealed a marked increase in parasite load at 24 h. Simultaneously, morphological changes in host cell membranes became apparent in bright-field images, suggesting biological and mechanical alterations likely associated with the progression of the infection.

**Figure 5:**
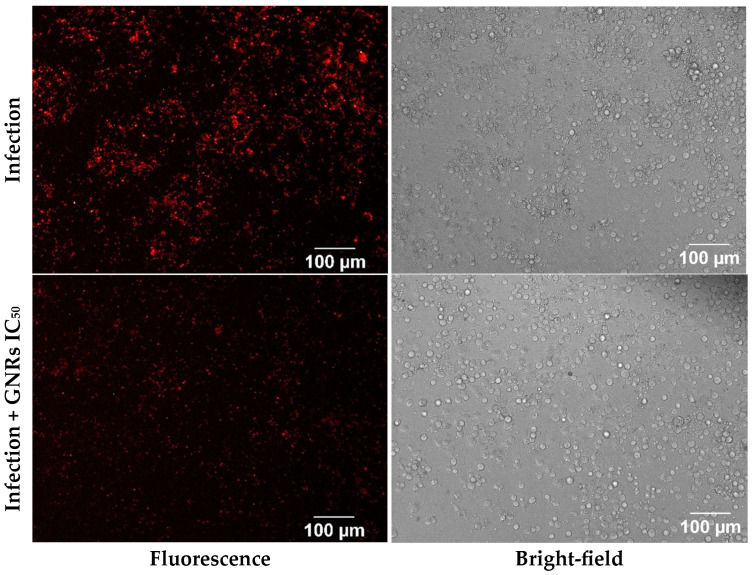
Fluorescence and bright-field images of infected THP-1 macrophages after 24 h. The upper row shows macrophages infected with *Leishmania* without GNRs. The bottom row shows infected macrophages treated for 24 h with GNRs at a IC_50_ concentration (2.8 μM Au or 4.2 pM GNRs). GNRs had an average length of 43±8 nm and an average diameter of 20±4 nm. In both rows, fluorescence images are shown on the left, and bright-field images are shown on the right.

This approach was also employed to assess the effect of gold nanorod treatment. As shown in Figure 5, the fluorescence intensity differed between the infected and treated groups. The bright-field images showed that treated cells remained viable and adherent, a finding further supported by biological assays [8]. These results suggest a potential antileishmanial effect of GNRs with low cytotoxicity toward host THP-1 cells.

**Figure 6:**
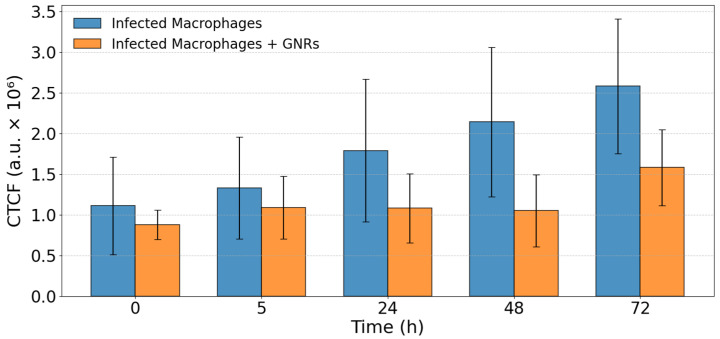
Evolution of CTCF in infected samples and samples treated with GNRs during a 72 h period.

This difference was quantified by mapping the fluorescence regions and calculating the CTCF (see Section 2.8) for three different samples in each case. Infected samples showed a clear progression of infection, whereas treated samples maintained relatively constant fluorescence levels for up to 48 h. Figure 6 presents the evolution of CTCF in infected and GNRs-treated samples during a 72 h period.

**Figure 7:**
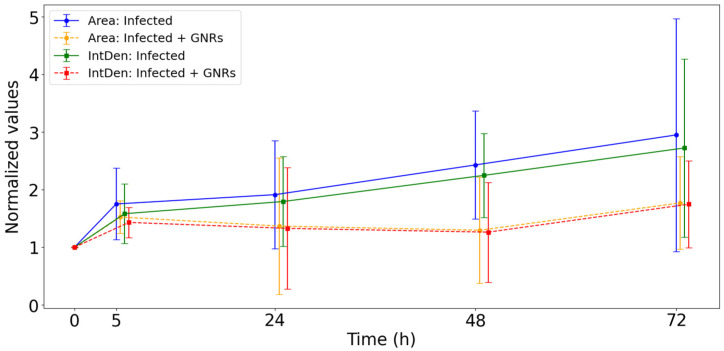
Evolution of mapped areas for the CTCF calculation and evolution of the Integrated Density (*Int Den*).

In addition, we separately evaluated the evolution of the infection areas and the fluorescence intensity, both of which contribute to the CTCF calculation (see Table S2 in Supplementary Materials). This analysis aimed to determine whether the observed reduction in CTCF in treated samples was driven primarily by a decrease in the infected area, a reduction in fluorescence intensity, or a combination of both. These separate analyses are presented in Figure 7, which illustrates the evolution of the mapped areas used for the CTCF calculation (see Figure S2 in Supplementary Materials), as well as the progression of fluorescence intensity (Integrated Density). The reduction in CTCF resulted from the combined decrease in both the infection areas and the fluorescence intensity.

### 3.3. Defocusing Microscopy Analysis

DM analysis included 118 healthy macrophages, 160 healthy macrophages exposed to GNRs, 110 macrophages infected with *Leishmania amazonensis*, and 135 infected macrophages incubated with GNRs. These groups were systematically analyzed to characterize the viscoelastic parameters of the cell membrane–cytoskeleton complex.

**Figure 8:**
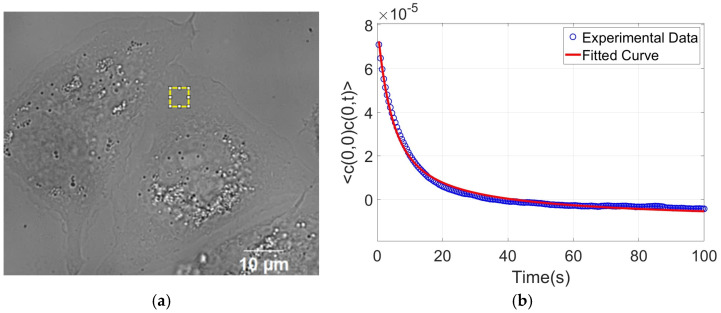
Representative examples of data analysis. (**a**) Example of a selected analysis area within the THP-1 macrophage. (**b**) Corresponding graph showing the CTAF curve obtained from the analysis. For this specific region, we obtained $k_{c}=(4.3\;\times 10^{-20}\pm \;4.2\;\times 10^{-22})\;J$ and η=(1.6±0.05) Pa·s.

For each cell, at least three distinct regions of the adhered cytoplasmic area were selected for analysis, explicitly excluding the nuclear region, since the nucleus contains organelles that absorb light and therefore, cannot be considered phase objects, potentially interfering with the measurements. The analysis consisted of evaluating the CTAF for each pixel within the selected region and averaging the results. Figure 8 presents an example of one such region analyzed, along with the corresponding averaged autocorrelation graph, which was used for the non-linear fitting of Equation (2) in MATLAB to obtain the values of the viscosity and bending modulus.

Based on statistical tests, we assessed the normality of the data and the significance of the observed differences. The Shapiro–Wilk test indicated that the data do not follow a normal distribution, thus requiring non-parametric methods. Therefore, the descriptive measures adopted for the parameters were the median and the IQR, as presented in the Table 1 below.

Given the non-parametric nature of the data, comparisons between pairs of independent samples were conducted using appropriate non-parametric tests, and the significance was evaluated through *p*-values. A detailed description of the complete statistical analysis is provided in the Tables S3–S5 in Supplementary Materials.

**Figure 9:**
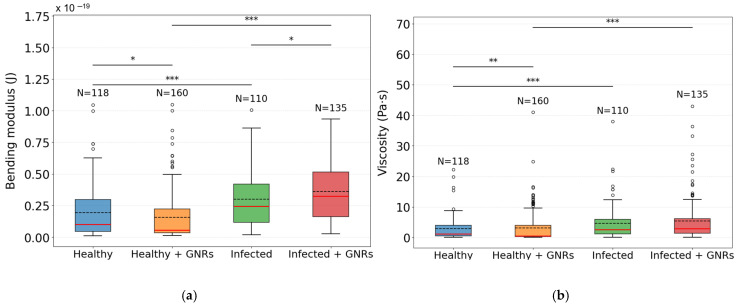
Boxplot representation of the bending modulus and viscosity parameters for the studied groups. (**a**) Boxplot for the bending modulus; (**b**) boxplot for the viscosity. The boxes indicate the interquartile range (IQR), and the horizontal line within each box represents the median. The whiskers extend to the minimum and maximum values within 1.5 times the IQR, while outliers are shown as individual points. Statistical comparisons were performed using the Mann–Whitney U test with the following sample sizes: Healthy (N = 118), Healthy + GNRs (N = 160), Infected (N = 110), and Infected + GNRs (N = 135). For $k_{c}$, the exact *p*-values were Healthy vs. Infected = $1.12\times 10^{-5}$, Healthy vs. Healthy + GNRs = $1.54\times 10^{-2}$, Infected vs. Infected + GNRs = $2.84\times 10^{-2}$, and Healthy + GNRs vs. Infected + GNRs = $1.17\times 10^{-18}$. For η, the exact *p*-values were Healthy vs. Infected = $5.71\times 10^{-4}$, Healthy vs. Healthy + GNRs = $1.16\times 10^{-3}$, Infected vs. Infected + GNRs = $4.72\times 10^{-1}$, and Healthy + GNRs vs. Infected + GNRs = $3.20\times 10^{-11}$ Graph Legend: 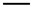 *p* value: *** *p* < 0.001; ** *p* < 0.01; * *p* < 0.05; - - - mean; 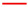 median; ○ out-liers; 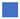 Healthy group; 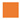 Healthy + GNRs group; 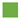 Infected group; 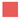 Infected + GNRs group.

To visualize and compare the distribution of the bending modulus and viscosity parameters between different groups, we represented the data using boxplot graphs (Figure 9). These graphs are useful for identifying the median, quartiles, outliers, and overall data dispersion, thus facilitating the comparison between samples.

Figure 9 shows the distribution of η and $k_{c}$ across the experimental groups. For the bending modulus, the Infected + GNRs group presented the highest median value $\left(3.24\times 10^{-20}J\right)$, followed by Infected $\left(2.46\times 10^{-20}\;J\right)$, while the Healthy and Healthy + GNRs groups showed lower values $\left(1.00\;\times \;10^{-20}\;J\;\mathrm{and}\;0.55\;\times \;10^{-20}\;J,\;respectively\right)$. All group comparisons were statistically significant (*p* < 0.05), as determined by the Mann–Whitney U test, indicating that both infection and GNR treatment affected the membrane–cytoskeleton $k_{c}$.

In terms of viscosity, the Healthy + GNRs group exhibited the lowest median value (0.57 Pa·s), followed by the Healthy group (1.23 Pa·s), while higher values were observed in the Infected (2.55 Pa·s) and Infected + GNRs (2.92 Pa·s) groups. The interquartile range was markedly broader in infected conditions (IQR ≈ 4.8), indicating greater variability. According to the Mann–Whitney U test, statistically significant differences were found between the Healthy and Infected groups (*p* < 0.001), Healthy and Healthy + GNRs groups (*p* < 0.01), and Healthy + GNRs and Infected + GNRs groups (*p* < 0.001). No significant differences were observed between the Infected and Infected + GNRs groups (*p* = 0.47).

## 4. Discussion

This study investigated the biomechanical responses of macrophages infected with *L. amazonensis* and treated with gold nanorods, with a particular focus on alterations in surface curvature (bending modulus) and intracellular viscosity. By integrating defocusing microscopy with fluorescence analysis, we correlated changes in parasitic load with mechanical modifications and nanoparticle interactions.

Through fluorescence analysis, the first notable observation was the small variation in CTCF levels in the treated samples, which may indicate that GNRs exerted a parasitostatic effect, preventing or delaying parasite proliferation, and thereby inhibiting population growth. This hypothesis is further supported by the observation that once the treatment is withdrawn at 24 h, parasite growth remains stagnant until becoming more pronounced after 72 h.

However, in biological assays [8], we observed that at higher concentrations of GNRs, such as CC_50_, nanorods exhibited a leishmanicidal effect. In contrast, at lower concentrations, such as those employed in our experiments, there may be a dynamic balance between parasite elimination and replication. In other words, the treatment can induce parasite death at a rate comparable to their replication, resulting in relatively stable fluorescence levels.

It is important to note that, at higher concentrations, the morphological stability of the nanorods becomes less reliable, which could compromise their therapeutic efficacy. This is attributable to the experimental protocol in which the surfactant responsible for maintaining the morphological stability of the nanorods is washed out to minimize its cytotoxic effects. As a result, the nanorods become more susceptible to aggregation, particularly at elevated concentrations. In contrast, lower concentrations favor the preservation of their structural integrity and colloidal stability while maintaining therapeutic activity.

A relevant characteristic of the RFP fluorescent protein is its ability to generate fluorescence without requiring cofactors or luminescent substrates. This property is advantageous because it prevents potential cell lysis induced by exogenous fluorophores and ensures that measurements remain free from external interference, preserving cell integrity, and enabling accurate, non-invasive monitoring through defocusing microscopy.

DM analysis revealed different viscoelastic changes in macrophages in response to *L. amazonensis* infection. The combined analysis of $k_{c}\;$ and η demonstrated that both infection and treatment significantly affect cell biomechanics. The determination of these parameters indicated that infection led to a significant rise: approximately three times in membrane $k_{c}$ and two-fold in η. These changes could be related to morphological and functional transformations associated with parasite establishment, including the formation of parasitophorous vacuoles, vesicle accumulation, and cytoskeletal reorganization [43,44].

Previous studies have shown that alterations in the membrane and cytoskeletal structure of cells can signal the presence of infections, triggering the production of cytokines and pro-inflammatory mediators. This process, in turn, attracts additional immune cells to the affected area or stimulates the formation of pseudopodia for phagocytosis. For example, a study using macrophages exposed to bacterial lipopolysaccharides demonstrated that membrane rigidity decreases, facilitating phagocytosis and suggesting an adaptive response to the presence of pathogenic agents [14].

On the other hand, another study related to atherosclerosis, a disease characterized by the accumulation of lipids, cholesterol, calcium, and other substances within the arteries, revealed that macrophages, upon exposure to oxidized low-density lipoproteins (oxLDLs), become stiffer and release pro-inflammatory substances that exacerbate the inflammatory process [45]. Therefore, a decrease in $k_{c}$ may suggest enhanced phagocytic activity in macrophages, while an increase may indicate infection and disease progression, as observed in our cells infected with Leishmaniasis.

The increase in η, in turn, can be attributed to several hypotheses. One possibility is reorganization of the cytoskeleton in response to infection, which may increase resistance to intracellular flow [43]. Another explanation is the accumulation of parasitic or inflammatory structures that interfere with fluid flow across the membrane. Furthermore, the presence of *Leishmania* can induce changes in cellular metabolism that increase viscosity, reflecting the attempt of the cell to restrict parasite spread through mechanical mechanisms [46,47].

In healthy macrophages, the median bending modulus and viscosity values reflect a baseline mechanical state consistent with the fluidity and deformability typical of resting cells. In the literature, the values for $k_{c}$ range from approximately $\sim 10^{-20}\;\mathrm{to}\;10^{-18}\;J$ [14,48], depending on the measurement technique and cell type. The viscosity values also vary according to the study technique, with ranges from approximately $\sim 10^{-3}\;\mathrm{to}\;10^{3}\;Pa\cdot s$ [16,48].

Specifically for macrophages, the values are concentrated around $\sim 10^{-19}\;J$ [14,48] for invasive techniques, such as optical tweezers. In our case, using DM, a non-invasive approach, lower values are expected, although still consistent with the expected range for living cells. The bending modulus of the healthy cells obtained in our study was $k_{c}=(1.00\times 10^{-20}\;\pm \;2.54\times 10^{-20})\;J$ and the viscosity η=(1.23±3.48)Pa·s, both reported as median ± IQR. These values lie within the predicted range for our membrane model and are consistent with those previously reported for cellular membranes and artificial lipid bilayers using different experimental approaches [48].

Treatment with GNRs resulted in approximately a two-fold reduction in both parameters, suggesting structural modulation potentially related to the internalization of nanomaterials and the resulting cytoskeletal rearrangement. The observed reduction may indicate a more fluid cellular state, possibly due to physical interactions between nanomaterials and the plasma membrane, together with alterations in cytoskeletal architecture [49,50]. Although no apparent functional impairment was observed, this modulation in non-activated cells underscores the need to consider mechanical effects even under non-pathological conditions.

GNRs can modulate the viscoelastic properties of the cell membrane through direct physical interactions and biochemical signaling. Upon adsorption or insertion into the lipid bilayer, they may locally alter membrane curvature, tension, and bending rigidity, thereby affecting lipid packing and intermonolayer friction. These perturbations can trigger cytoskeletal remodeling, particularly actin filament reorganization, which further influences cell stiffness and viscosity. Moreover, GNRs binding may promote the clustering of membrane proteins, such as integrins, altering mechanotransduction pathways and contributing to the observed mechanical phenotype. Together, these processes lead to measurable changes in membrane elasticity and viscosity, as reported in studies that combine optical tracking of GNRs with cell mechanics assays [51,52].

These mechanistic effects are in agreement with previous studies showing that gold nanoparticles, at non-toxic concentrations, interact with healthy cells while maintaining their viability, inducing slight alterations in cytoskeletal organization that reduce stiffness. In contrast, at toxic concentrations, nanorods increase cell stiffness [53,54]. Our findings of decreased bending modulus are consistent with the expected effects of non-toxic nanoparticle interactions, promoting a state of reduced stiffness in healthy cells, which is essential for normal cellular function and regulatory processes.

Interestingly, treatment of infected cells with GNRs resulted in a further increase in the bending modulus, without significantly affecting viscosity compared to the untreated infected group. This suggests that GNRs improve cell stiffness, possibly by reinforcing the cytoskeletal structure or directly influencing the organization [49]. The lack of a change in viscosity may indicate that infection already induces a saturated mechanical state in terms of intracellular fluidity, without an additional impact of treatment.

Overall, the data indicate that GNRs modulate the viscoelastic properties of macrophages in a manner dependent on the cellular state. In healthy cells, they appear to promote mild destabilization, whereas in infected cells, they reinforce structural rigidity. These findings are relevant both for understanding the interaction between nanomaterials and host cells and for guiding the development of therapeutic strategies involving GNRs in the context of leishmaniasis. More studies are needed to determine whether these mechanical alterations correlate with functional outcomes, such as phagocytic capacity or parasite clearance.

## 5. Conclusions

In this study, defocusing microscopy and fluorescence imaging were used to investigate the progression of *Leishmania* infection in macrophages, as well as the biomechanical alterations induced by gold nanorods, with the aim of evaluating their therapeutic potential in the treatment of leishmaniasis.

The data collected provided a comprehensive characterization of the distribution of the bending modulus and viscosity under distinct experimental conditions. The interaction between macrophages and Leishmania parasites resulted in a marked increase in both viscoelastic parameters, establishing a biomechanical signature that may serve as a valuable biomarker for monitoring infection progression and membrane remodeling. These modifications may be interpreted as potentially beneficial, as they could strengthen immune function and the ability to respond to the parasite. However, we acknowledge that such interpretations should be made with caution, since direct functional correlations between mechanical remodeling and antiparasitic activity still need to be established. Future studies will aim to clarify whether these biomechanical modulations directly contribute to leishmanicidal activity.

Notably, treatment with GNRs modulated these viscoelastic properties in a context-dependent manner. In healthy cells, it induced a reduction in both the bending modulus and viscosity, consistent with a state of increased membrane fluidity. Conversely, in infected cells, GNRs increased the bending modulus without significantly altering the viscosity, suggesting a reinforcement of cellular structure without further impact on cytoplasmic flow.

In addition to mechanical modulation, GNRs appeared to exert a leishmanicidal effect, as evidenced by the stability of the fluorescence intensity and delayed parasite proliferation after treatment. These findings support the idea that GNRs interact non-toxically with macrophage membranes and the cytoskeleton and may modulate viscoelastic responses that contribute to infection containment.

Although direct comparisons of GNRs with conventional drugs, such as sodium stibogluconate, meglumine antimoniate, and amphotericin B, are currently limited due to the preclinical stage of GNRs development, GNRs have demonstrated leishmanicidal activity at much lower concentrations than these standard treatments. This indicates potential for greater long-term cost effectiveness, not only by reducing the required dose but also by lowering indirect costs associated with hospitalization, management of adverse effects, and discontinuation of treatment. Although more translational studies are needed, our findings support the view that GNRs-based therapies may, in the future, represent a more cost-effective alternative.

Our results underscore the utility of defocusing microscopy, combined with the developed viscoelastic model, as a powerful and non-invasive approach to quantifying biomechanical properties in live cell systems. This methodology can be extended to the investigation of other pathological conditions, host–pathogen interactions, and drug screening platforms.

Furthermore, the mechanical profiles described here provide a valuable framework for evaluating future therapeutic strategies, including the integration of photothermal approaches involving GNRs and laser irradiation. Together, these insights pave the way for the rational design of innovative biomedical strategies targeting infectious diseases.

## Figures and Tables

**Table 1:** Descriptive values of bending modulus ($k_{c}$) and viscosity (η) of the experimental groups.

$k_{c}$ ** (J)**	**Median × 10^−20^**	**IQR × 10^−20^**
Healthy	1.00	2.54
Infected	2.46	3.02
Healthy + GNRs	0.55	1.86
Infected + GNRs	3.24	3.53

η ** (Pa·s)**	**Median**	**IQR**
Healthy	1.23	3.48
Infected	2.55	4.77
Healthy + GNRs	0.57	3.79
Infected + GNRs	2.92	4.84

## Data Availability

Data are contained within the article or Supplementary Materials.

## Supplementary Materials

The following supporting information can be downloaded at https://www.mdpi.com/article/10.3390/nano15171373/s1, Figure S1: Inverted microscope Nikon Eclipse Ti-E setup; Figure S2: Representative images illustrating the fluorescence microscopy analysis and quantification methodology. (a) Fluorescence image of an infected macrophage sample, showing intracellular parasites labeled with fluorescence. (b) Example of fluorescence quantification using ImageJ: mapping of fluorescence-positive regions (regions of interest) for corrected total cell fluorescence (CTCF) calculation.; Table S1: Quantification of total fluorescence intensity (CTCF) at different time points; Table S2: Time-course analysis of normalized cell area and integrated density (*IntDen*); Table S3: Results of the Mann–Whitney U test applied to the bending modulus ($k_{c}$) and viscosity (η) across the experimental groups; Table S4: Results of the Shapiro–Wilk normality test for the bending modulus ($k_{c}$) and viscosity (η) in each experimental group; Table S5: Descriptive statistics (mean, median, standard deviation, and interquartile range) for the bending modulus ($k_{c}$) and viscosity (η) of the experimental groups; Text S1: Detailed mathematical derivation of the contrast temporal autocorrelation functions; Figure S3: Position and wave vectors in real space (a and reciprocal space (b), showing their respective components and vector representations; Figure S4: Geometry of the electric field propagation through a cell interface. Caption: $E_{0}:$ incident electric field; $h\left(\vec{\rho }\right)$ is the amplitude of the undulation of the wavy interface; $n_{1}$ and $n_{2}$ are the refractive indices of the medium before and after the interface. Also shown as Figure 1a in the main manuscript; Figure S5: Geometry of the electric field propagation through the defocused microscope. References [25,38,39,40] are cited in the supplementary materials.

## Abbreviations

The following abbreviations are used in this manuscript:

AFM	Atomic Force Microscopy
AgNO_3_	Silver nitrate
AR	Aspect ratio
ATCC	American Type Culture Collection
CL	Cutaneous Leishmaniasis
CTAB	Cetyltrimethylammonium bromide
CTAFs	Contrast temporal autocorrelation functions
CTCF	Corrected total cell fluorescence
DM	Defocusing microscopy
DMSO	Dimethyl sulfoxide
GNRs	Gold nanorods
HAuCl_4_	Chloroauric acid
*IntDen*	Integrated Density
IQR	Interquartile range
Kc	Bending modulus
M∅s	Macrophages
NA	Numerical aperture
NaBH_4_	Sodium borohydride
oxLDLs	Oxidized low-density lipoproteins
PFS	Perfect Focus System
PMA	Phorbol 12-myristate 13-acetate
RFP	Red fluorescent protein
SI	Selectivity index
SPR	Surface plasmon resonance
TEM	Transmission electron microscopy
η	Viscosity
WHO	World Health Organization
