# Supplementary material for: Investigation of Viscoelastic Properties of Macrophage Membrane–Cytoskeleton Induced by Gold Nanorods in Leishmania Infection
Source: Nanomaterials (Basel). 2025 Sep 5;15(17):1373. doi: 10.3390/nano15171373 (PMC12430347; doi:10.3390/nano15171373)
Supplement: Supplementary file 1 [file nanomaterials-15-01373-s001.zip › nanomaterials-3794814-supplementary.pdf]

nanomaterials

MDPI

Supplementary Materials

Investigation of Viscoelastic Properties of Macrophage Membrane–Cytoskeleton Induced by Gold Nanorods in Leishmania Infection

Maria L. B. Pertence [1], Marina V. Guedes [1], Rosimeire C. Barcelos [2], Jeronimo N. Rugani [3], Rodrigo P. Soares [3], Joyce L. V. Cruz [3], Alessandra M. de Sousa [3], Rubens L. do Monte-Neto [3], Livia G. Siman [1], Anna C. P. Lage [3,*] and Ubirajara Agero [1,*]

[1] Department of Physics, Federal University of Minas Gerais, Av. Antônio Carlos 6627, Pampulha, Belo Horizonte 31270-901, MG, Brazil; mpertence@fis.dout.ufmg.br (M.L.B.P.); marinavguedes@ufmg.br (M.V.G.); liviasg@fisica.ufmg.br (L.G.S.)

[2] Department of Chemistry, Federal University of São João del-Rei, Praça Dom Helvécio 74, Centro, São João del-Rei 36301-160, MG, Brazil; rosicbarcelos@ufsj.edu.br (R.C.B.)

[3] René Rachou Institute, Oswaldo Cruz Foundation, Av. Augusto de Lima 1715, Barro Preto, Belo Horizonte 30190-002, MG, Brazil; jeronimomnr@hotmail.com (J.N.R.); rodrigo.pedro@fiocruz.br (R.P.S.); joycevianac@gmail.com (J.L.V.C.); alessandra.sousa@fiocruz.br (A.M.d.S.); rubens.monte@fiocruz.br (R.L.d.M.-N.)

* Correspondence: anna.lage@fiocruz.br (A.C.P.L.); bira@ufmg.br (U.A.)

Academic Editor: Sónia Carabineiro

Received: 17 July 2025
Revised: 22 August 2025
Accepted: 26 August 2025
Published: 5 September 2025

Citation: Pertence, M.L.B.; Guedes, M.V.; Barcelos, R.C.; Rugani, J.N.; Soares, R.P.; Cruz, J.L.V.; de Sousa, A.M.; do Monte-Neto, R.L.; Siman, L.G.; Lage, A.C.P.; et al. Investigation of Viscoelastic Properties of Macrophage Membrane-Cytoskeleton Induced by Gold Nanorods in Leishmania Infection. *Nanomaterials* **2025**, *15*, 1373. https://doi.org/10.3390/nano15171373

Copyright: © 2025 by the author. Licensee MDPI, Basel, Switzerland. This article is an open access article distributed under the terms and conditions of the Creative Commons Attribution (CC BY) license (https://creativecommons.org/licenses/by/4.0/).

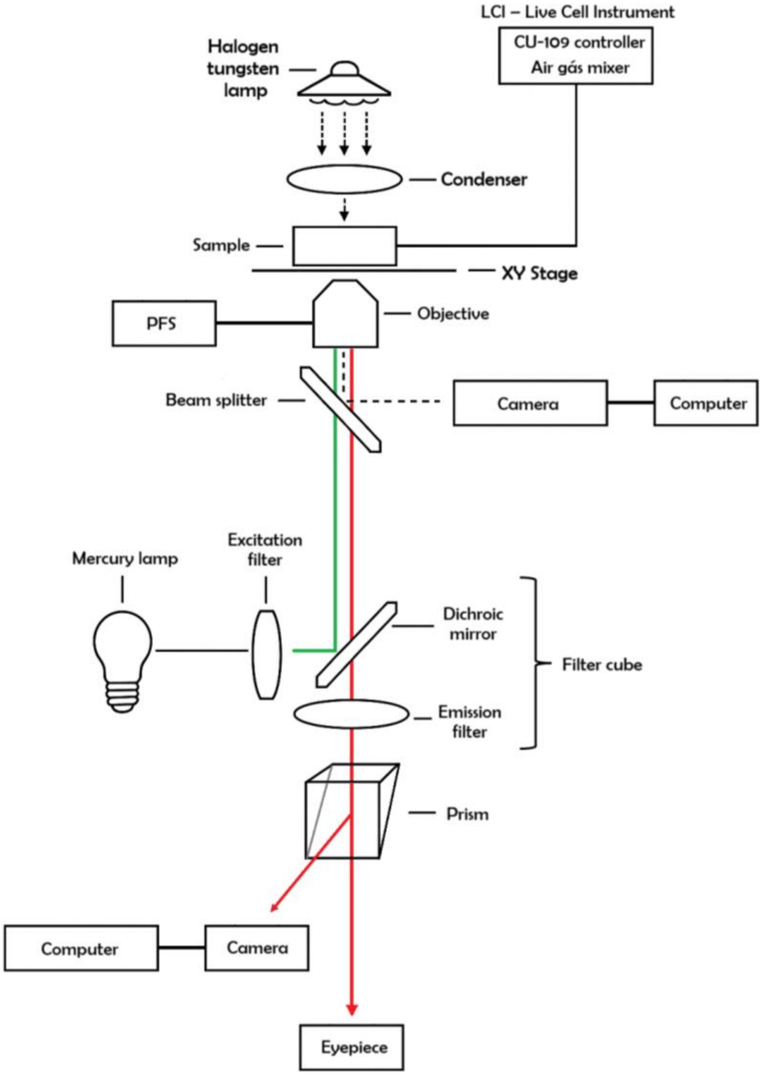

Figure S1. Inverted microscope Nikon Eclipse Ti-E setup.

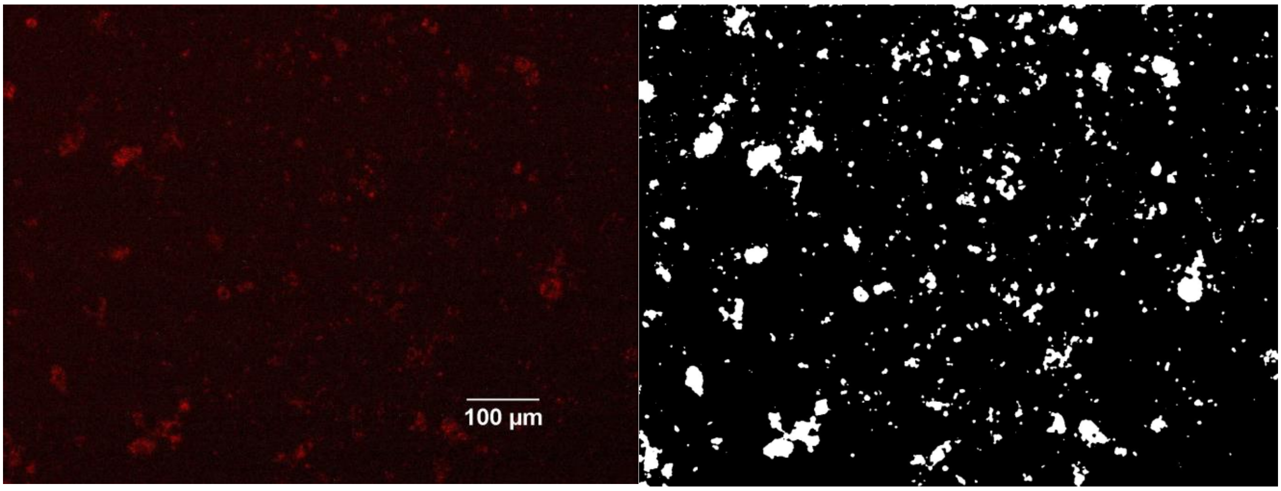

(a) **(b)**

Figure S2. Representative images illustrating the fluorescence microscopy analysis and quantification methodology. (**a**) Fluorescence image of an infected macrophage sample, showing intracellular parasites labeled with fluorescence. (**b**) Example of fluorescence quantification using ImageJ:

mapping of fluorescence-positive regions (regions of interest) for corrected total cell fluorescence (CTCF) calculation.

Table S1. Quantification of total fluorescence intensity (CTCF) at different time points.

Time (h)	CTCF Infected cells	Standard Deviation Infected cells	CTCF Infected cells + GNRs	Standard Deviation Infected cells + GNRs
0	1.11E+06	6.01E+05	8.77E+05	1.79E+05
5	1.33E+06	6.27E+05	1.09E+06	3.85E+05
24	1.79E+06	8.79E+05	1.08E+06	4.25E+05
48	2.14E+06	9.16E+05	1.05E+06	4.44E+05
72	2.58E+06	8.29E+05	1.58E+06	4.67E+05

Table S2. Time-course analysis of normalized cell area and integrated density (*IntDen*).

Tempo (h)	Normalized Area Infected cells	Normalized Area Infected + GNRs	Normalized *IntDen* Infected cells	Normalized *IntDen* Infected cells + GNRs
0	1.00	1.00	1.00	1.00
5	1.54	1.58	1.43	1.48
24	1.64	1.13	1.60	1.14
48	2.07	1.11	1.99	1.17
72	2.16	1.66	2.14	1.65

Table S3. Results of the Mann–Whitney U test applied to the bending modulus (k_c) and viscosity (η) across the experimental groups.

Groups k_c	Mann-Whitney (p-value)
Healthy vs Infected	1.12E-05
Healthy vs Healthy + GNRs	1.54E-02
Infected vs Infected + GNRs	2.84E-02
Healthy + GNR vs Infected + GNRs	1.17E-18

Groups η	Mann-Whitney (p-value)
Healthy vs Infected	5.71E-04
Healthy vs Healthy + GNRs	1.16E-03
Infected vs Infected + GNRs	4.72E-01
Healthy + GNR vs Infected + GNRs	3.20E-11

Table S4. Results of the Shapiro–Wilk normality test for the bending modulus (k_c) and viscosity (η) in each experimental group.

Groups η	Shapiro-Wilk (p-value)
Healthy	1.56E-14
Infected	1.96E-13
Healthy + GNRs	4.19E-19
Infected + GNRs	3.36E-16

Groups k_c	Shapiro-Wilk (p-value)
Healthy	9.41E-12
Infected	1.00E+00
Healthy + GNRs	9.37E-17
Infected + GNRs	1.00E+00

Table S5. Descriptive statistics (mean, median, standard deviation, and interquartile range) for the bending modulus (k_c) and viscosity (η) of the experimental groups.

Groups η	Mean	Median	Standart deviation	IQR
Healthy	2.93	1.23	3.83	3.48

Healthy + GNRs	3.13	0.57	5.39	3.79
Infected	4.63	2.55	5.52	4.77
Infected + GNRs	5.45	2.92	7.10	4.84

**Groups k_c**	**Mean**	**Median**	**Standart deviation**	**IQR**
Healthy	1.93E-20	9.99E-21	2.06E-20	2.54E-20
Healthy + GNRs	1.56E-20	5.52E-21	1.97E-20	1.86E-20
Infected	3.01E-20	2.46E-20	2.26E-20	3.02E-20
Infected + GNRs	3.61E-20	3.24E-20	2.35E-20	3.53E-20

Text S1. Detailed mathematical derivation of the contrast temporal autocorrelation functions

To interpret the propagation of the electric field and its angular spectrum in a microscope operating in bright field mode, we consider that the electric field propagates along the direction perpendicular to the wave vector $\vec{k}$, as illustrated in the reference frame below. In this system, $\vec{\rho}$ represents the position vector in the (x, y) plane, $\vec{q}$ is the corresponding wave vector in the plane, and the light propagates along the z-axis.

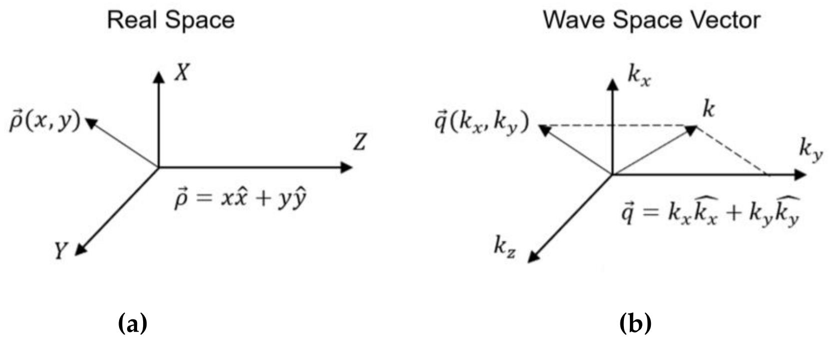

Figure S3. Position and wave vectors in real space (a and reciprocal space (b), showing their respective components and vector representations.

The electric field can be described as a scalar field, assuming a fixed linear polarization and a time dependence of the form $e^{-i\omega t}$:

$$E(\vec{r}, t) = E_0\, e^{-i(\omega t - \vec{k} \cdot \vec{r})} \tag{S1}$$

Using the Fourier transform defined by:

$$\mathcal{F}\{g(\vec{\rho})\} = G(\vec{q}) = \int g(\vec{\rho}) e^{-i\vec{q}\cdot\vec{\rho}} d\vec{\rho} \tag{S2}$$

Where $\int d\vec{\rho} = \int_{-\infty}^{+\infty} dx = \int_{-\infty}^{+\infty} dy$. And its inverse transform is given by:

$$\mathcal{F}^{-1}\{G(\vec{q})\} = g(\vec{\rho}) = \frac{1}{(2\pi)^2} \int G(\vec{q}) e^{-i\vec{q}\cdot\vec{\rho}} d\vec{q} \tag{S3}$$

Where $\int d\vec{q} = \int_{-\infty}^{+\infty} d\vec{q}_x = \int_{-\infty}^{+\infty} d\vec{q}_y$. Thus, the electric field can be written as a Fourier transform in the (x, y) plane, according to the formalism for the propagation of the angular spectrum [1,2]:

$$E(\vec{\rho}, z) = \frac{1}{(2\pi)^2} \int A(\vec{q}, z)\, e^{i\vec{q}\cdot\vec{\rho}} d\vec{q} \tag{S4}$$

In this equation, $A(\vec{q}, z)$ represents the angular spectrum, which can be obtained through the inverse transform:

$$A(\vec{q}, z) = \int E(\vec{\rho}, z)\, e^{-i\vec{q}\cdot\vec{\rho}} d\vec{\rho} \tag{S5}$$

Based on the coordinate system described earlier, it is possible to analyze the propagation of the electric field in the microscope through angular decomposition. According to the references [3,4], the propagation of the total diffracted angular spectrum can be treated as a superposition of the spectra $A(\vec{q}, z)$ for the different wave vectors $\vec{q}$, assuming the paraxial approximation, which is equivalent to the Fresnel approximation for diffraction.

When light passes through an object of phase, as illustrated in Figure S4, the incident electric field undergoes diffraction and is described by $\overrightarrow{E_0} = E_0 e^{i\varphi\overrightarrow{(\rho)}}$, where the phase $\varphi\overrightarrow{(\rho)} = \Delta n k_0 h\overrightarrow{(\rho)}$. After propagation through the optical system of a microscope in bright field mode, the electric field in the image plane can be represented by:

$$E_I = C E_0 e^{i\varphi\overrightarrow{(\rho)}} \tag{S6}$$

In this expression, $\Delta n = n_2 - n_1$ represents the difference in refractive index between the phase object and the medium, $h\overrightarrow{(\rho)}$ is the interface ripple profile of the object, k_0 is the wave number of light in vacuum, and E_0 is the field without interface in free space, assumed to have constant amplitude and phase.

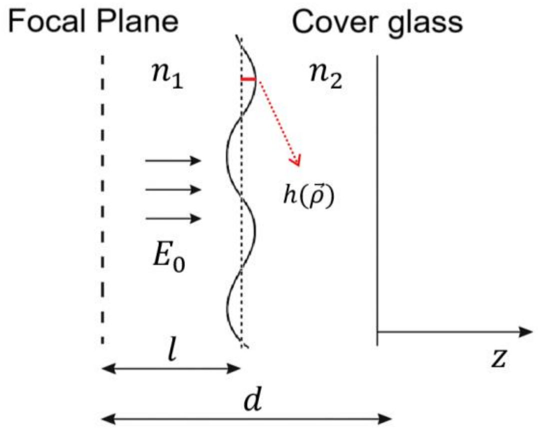

Figure S4. Geometry of the electric field propagation through a cell interface. Caption: E_0: incident electric field; $h(\vec{\rho})$ is the amplitude of the undulation of the wavy interface; n_1 and n_2 are the refractive indices of the medium before and after the interface. Also shown as Figure 1(**a**) in the main manuscript.

The intensity of light is defined as $I = |E(\vec{\rho})|^2$, which results in:

$$I = C^2 E_0^{2e^{2i\varphi(\vec{\rho})}} \tag{S7}$$

The contrast, in turn, is given by:

$$C(\vec{\rho}) = \frac{|E(\vec{\rho})|^2 - E_0{}^2}{|E_0|^2} \tag{S8}$$

If the phase object introduces only a phase modulation, without altering the amplitude of the field, the intensity remains constant and the contrast is zero. However, when the phase difference $\varphi(\vec{\rho})$ is small, it is possible to expand the electric field expression in a Taylor series, keeping only the first-order term in $h(\vec{\rho})$, that is, assuming $\varphi(\vec{\rho}) \ll 1$. In this case, the electric field can be approximated by:

$$\vec{E} \approx E_0[1 - i\varphi(\vec{\rho})] = E_0\,[1 + i\Delta n k_0 h(\vec{\rho})] \tag{S9}$$

In this small phase regime, the total electric field is composed of two terms: the transmitted field E_0, and the diffracted field, given by $iE_0\varphi(\vec{\rho})$, which is out of phase by $\frac{\pi}{2}$ with respect to the transmitted field. Since the two fields are in $\frac{\pi}{2}$ out-of-phase, the interference between them does not produce intensity variations, maintaining the contrast at zero.

However, by intentionally introducing an additional phase shift of $\pm\frac{\pi}{2}$ in the transmitted field, the interference with the diffracted field becomes either constructive or destructive, producing intensity variations proportional to the phase of the object. In this configuration, the electric field in the image plane can be approximated by:

$$E_I \approx iE_0[\pm 1 - \varphi(\vec{\rho})] \tag{S10}$$

Therefore, with this additional phase shift, the intensity is no longer constant and begins to depend on the phase of the object. In the first order of $\varphi(\vec{\rho})$, the intensity starts to reflect variations that were previously invisible in the purely phase regime:

$$I(\vec{\rho}) = |E_0|^2[1 \pm 2\varphi(\vec{\rho})] \tag{S11}$$

Consequently, the contrast is given by:

$$C(\vec{\rho}) = \frac{|E(\vec{\rho})|^2 - E_0{}^2}{|E_0|^2} = 2\varphi(\vec{\rho}) \tag{S12}$$

Thus, the phase object becomes visible, and the contrast is proportional to the thickness of the object, in the limit where $\varphi = \Delta n k_0 h \ll 1$. In a defocused inverted microscope, as illustrated in Figure S5:

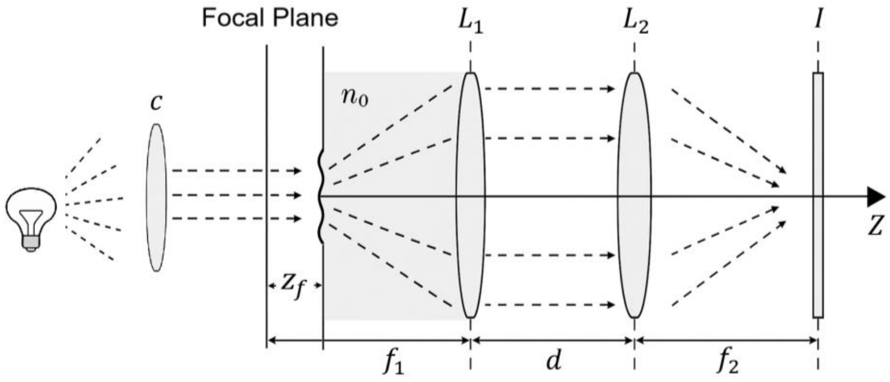

Figure S5. Geometry of the electric field propagation through the defocused microscope. Caption: C: Condenser; L_1: Objective; L_2: Tube lenz. z_f: Defocused amount. Also shown as Figure 1(**b**) in the main manuscript.

In the absence of a phase object, the phase of the electric field at a distance d in a medium with refractive index n_1 would be simply $\varphi_0 = n_1 k_0 d$. With the presence of the phase object, this phase is modified, and the new expression becomes:

$$\varphi_1 = n_1 k_0 (l + h) + n_2 k_0 [d - (l + h)] \tag{S13}$$

Therefore, the phase difference $\Delta\varphi = \varphi_1 - \varphi_0$:

$$\begin{aligned} \Delta\varphi = \varphi_1 - \varphi_0 &= (n_1 - n_2) k_0 (l - d) + (n_1 - n_2) k_0 h \\ &= -\Delta n k_0 (l - d) - \Delta n k_0 h \end{aligned} \tag{S14}$$

Which can be decomposed as:

$$\Delta\varphi = constante + \varphi(\vec{\rho}) \tag{S15}$$

The roughness $h(\vec{\rho})$ of the phase object's interface can be decomposed into a Fourier series, similarly to the electric field, as:

$$h(\vec{\rho}) = \frac{1}{\sqrt{S}} \sum_{\overrightarrow{q'}} h(\overrightarrow{q'})\, sen(\overrightarrow{q'}.\ \vec{\rho}) \tag{S16}$$

Here, $h(\vec{q}')$ represents the amplitude of the $\vec{q}'$ mode of the undulation, and S is the surface of the interface. For $h(\vec{\rho})$ to be real, the necessary condition is that $h(\overrightarrow{q'})$ also be real. Expanding this expression, the scalar field resulting from the diffraction of light by the object is given by:

$$E(\vec{\rho}, 0) = E_0 \left[1 + \frac{i \Delta n k_0}{\sqrt{S}} \sum_{\overrightarrow{q'}} h(\overrightarrow{q'})\, sen(\overrightarrow{q'}.\ \vec{\rho}) \right] \tag{S17}$$

The corresponding angular spectrum is given by:

$$A_0 = \int E(\vec{\rho}, 0) e^{-\vec{q}\cdot\vec{\rho}}\ d\vec{\rho} \tag{S18}$$

By changing the variable $\overrightarrow{q'} \rightarrow \vec{q}$, the intensity of the observed light can be calculated, defined by $I(\vec{\rho})\ \alpha\ |E(\vec{\rho})|^2$, resulting in:

$$I(\vec{\rho}) = I_0 \left\{ 1\ \frac{2 \Delta n k_0}{\sqrt{S}} \sum_{\vec{q}} h(\vec{q})\, sen(\vec{q}.\vec{\rho})\, sen\left(\frac{z_f}{2k} q^2 \right) \right\} \tag{S19}$$

In a similar way, using the definition (1.7), we obtain the image contrast:

$$C(\vec{\rho}) = \frac{2 \Delta n k_0}{\sqrt{S}} \sum_{\vec{q}} \left[h(\vec{q})\, sen(\vec{q}.\vec{\rho})\, sen\left(\frac{z_f}{2k} q^2 \right) \right] \tag{S20}$$

Our phase object consists of an adhered interface and a free one, allowing height fluctuation and membrane movement. In this case, the height profile is defined as the sum of a time-independent part and a time-dependent part, in real space, as described below:

$$H(\vec{\rho},t) = h(\vec{\rho}) + u(\vec{\rho},t) \tag{S21}$$

The time average of the height profile is given by $\langle H(\vec{\rho},t)\rangle = h(\vec{\rho})$, since, mathematically, $\langle u(\vec{\rho},t)\rangle = 0$. Correspondingly, in wave vector space, the height profile is given by:

$$H(\vec{q},t) = h(\vec{q}) + u(\vec{q},t) \tag{S22}$$

We can then substitute the height $h(\vec{\rho})$ with $H(\vec{\rho},t)$ in the expression for contrast, obtaining its time-dependent form:

$$C(\vec{\rho},t) = \frac{2\Delta n k_0}{\sqrt{S}} \sum\nolimits_{\vec{q}} H(\vec{q},t)\ sen(\vec{q}\cdot\vec{\rho})\, sen\left(\frac{z_f q^2}{2k}\right) \tag{S23}$$

The time average of the contrast is, therefore:

$$\begin{aligned}\langle C(\vec{\rho},t)\rangle &= \frac{2\Delta n k_0}{\sqrt{S}} \sum\nolimits_{\vec{q}} \langle H(\vec{q},t)\rangle\ sen(\vec{q}\cdot\vec{\rho})\, sen\left(\frac{z_f q^2}{2k}\right) \\ &= \frac{2\Delta n k_0}{\sqrt{S}} \sum\nolimits_{\vec{q}} h(\vec{q})\ sen(\vec{q}\cdot\vec{\rho})\, sen\left(\frac{z_f q^2}{2k}\right)\end{aligned} \tag{S24}$$

We define the fluctuation of the contrast as:

$$\Delta C(\vec{\rho},t) = C(\vec{\rho},t) - \langle C(\vec{\rho},t)\rangle \tag{S25}$$

Substituting the previous expressions, we obtain:

$$\begin{aligned}\Delta C(\vec{\rho},t) &= \frac{2\Delta n k_0}{\sqrt{S}} \sum\nolimits_{\vec{q}} \{H(\vec{q},t) - h(\vec{q})\}\ sen(\vec{q}\cdot\vec{\rho})\, sen\left(\frac{z_f q^2}{2k}\right) \\ &= \frac{2\Delta n k_0}{\sqrt{S}} \sum\nolimits_{\vec{q}} u(\vec{q},t)\ sen(\vec{q}\cdot\vec{\rho})\, sen\left(\frac{z_f q^2}{2k}\right)\end{aligned} \tag{S25}$$

The objective is to compute the average of the contrast fluctuation for a stationary system, defined by:

$$\langle \Delta C(\vec{\rho},0)\, \Delta C(\vec{\rho} + \vec{r},t)\rangle \tag{S26}$$

Using the previous equation, we have:

$$\begin{gathered}\Delta C(\vec{\rho} + \vec{r},t) = \\ \frac{2\Delta n k_0}{\sqrt{S}} \sum\nolimits_{\vec{q}} u(\vec{q},t)\ sen(\vec{q} + (\vec{\rho} + \vec{r}))\, sen\left(\frac{z_f q^2}{2k}\right)\end{gathered} \tag{S27}$$

Substituting (S26) and (S28) into the expression for the average of the contrast fluctuation, we obtain:

$$= \langle \frac{(2\Delta n k_0)^2}{S} \sum_{\vec{q}} \sum_{\vec{q'}} u(\vec{q},0) u^*(\overrightarrow{q'},t)\, sen\left(\frac{z_f q^2}{2k}\right) sen\left(\frac{z_f q'^2}{2k}\right) \times sen(\vec{q} \cdot \vec{\rho})\; sen\left(\overrightarrow{q'} \cdot (\vec{\rho} + \vec{r})\right) \rangle \tag{S28}$$

Initially, we analyze separately the mean value of the sinusoidal functions present in the expression. By applying trigonometric identities to equation (S29), we can rewrite this part as:

$$\langle sen(\overrightarrow{q}.\vec{\rho})\; sen(\overrightarrow{q'}.(\vec{\rho} + \vec{r})) \rangle = \delta_{\vec{q}\vec{q}} \left(\frac{\cos(\vec{q} \cdot \vec{r})}{2}\right) \tag{S30}$$

Furthermore, for a stationary system with simple temporal relaxation, we have:

$$< u(\vec{q},0) u^*(\vec{q},t) > = |u(\vec{q},t)|^2\, e^{-\omega(\vec{q})t} \tag{S29}$$

Substituting these relationships, the expression for the temporal correlation of the contrast fluctuation reduces to:

$$\langle \Delta C(\vec{\rho},0)\, \Delta C(\vec{\rho} + \vec{r}, t) \rangle = \frac{2(\Delta n k_0)^2}{S} \sum_{\vec{q}} |u(\vec{q},t)|^2\, e^{-\omega(\vec{q})t}\; sen^2\left(\frac{z_f q^2}{2k}\right) (\cos(\vec{q} \cdot \vec{r}) \tag{S32}$$

With this result, we can observe that, for small defocuses and small wave vectors $\vec{q}$, the approximation holds:

$$\frac{z_f}{2k} q^2 \ll 1 \tag{S30}$$

Thus

$$sen^2\left(\frac{z_f}{2k} q^2\right) \approx \left(\frac{z_f}{2k} q^2\right)^2 \tag{S31}$$

For the determination of the viscoelastic parameters of cell membranes, we interpret the autocorrelation function as a purely temporal function, disregarding spatial variations. Therefore, in the above equation, we take $\vec{r} = 0$, resulting in a temporal autocorrelation function that does not depend on $\vec{\rho}$:

$$< \Delta C\,(0,0) \Delta C(0,t) > = \frac{2(\Delta n k_0)^2}{S} \sum_{\vec{q}} |u(\vec{q})|^2\, e^{-\omega(q)t} \left(\frac{z_f}{2k} q^2\right)^2 \tag{S32}$$

In the continuous case, the sum over the modes $\vec{q}$ becomes an integral in the two-dimensional Fourier space:

$$\sum_{\vec{q}} \rightarrow \frac{S}{(2\pi)^2} \int_0^{q_{max}} d\vec{q} \tag{S33}$$

Where $d\vec{q} = qdqd\theta$ e q_{max} is the largest wave vector collected by the microscope objective with numerical aperture NA,. Thus, the contrast autocorrelation function in the continuous regime can be rewritten as:

$$= \frac{2(\Delta n k_0)^2}{(2\pi)^2} \int_0^{q_{max}} q d\vec{q} \int_0^{2\pi} |u(\vec{q})|^2 \, e^{-\omega(q)t} \left(\frac{z_f^2 \, q^4}{4k^2}\right) d\theta \tag{S34}$$

The form of $|u(\vec{q})|^2$ arises from the discussion presented in [4] on thermal fluctuations in surfaces. These fluctuations are influenced by gravitational potential energy, surface tension, and a confinement potential, resulting in the spatial potential spectrum:

$$\langle|h(\vec{q})|\rangle^2 = \frac{k_b T}{k_c q^4 + \sigma q^2 + \gamma} \tag{S35}$$

This same expression is adopted in the study of the coupling between the membrane and the cytoskeleton, leading to a modified form:

$$\langle|u(\vec{q})|^2\rangle = \frac{k_b \, T_{ef}}{k_c q^4 + \sigma_{ef} q^2 + \gamma} \tag{S39}$$

In the denominator, the term in q4 represents the contribution from curvature fluctuations, with k_c being the bending modulus of the membrane. The additional terms incorporate effects from the cytoskeleton: σ_{ef} is the effective surface tension introduced by the membrane's connections to the cytoskeleton, while γ represents a confinement potential related to the shear modulus. The effective temperature T_{ef} reflects the non-equilibrium conditions that may be present.

At this point, we are primarily interested in the contributions from k_c associated with the morphological changes of the membrane. For this, we adopt a model in which the membrane is considered free, that is, without confinement or surface tension, which corresponds to taking the limit $d \rightarrow \infty$ e $\sigma_{ef} = \gamma = 0$. In this case, the power spectrum simplifies to:

$$\langle|u(\vec{q})|^2\rangle = \frac{k_b T}{k_c q^4} \tag{S40}$$

Substituting this form into the autocorrelation expression, we obtain:

$$= \frac{2(\Delta n k_0)^2}{(2\pi)^2} \int_0^{q_{max}} q d\vec{q} \int_0^{2\pi} \left(\frac{k_b T}{k_c q^4}\right) e^{-\omega(\vec{q})t} \left(\frac{z_f^2 \, q^4}{4k^2}\right) d\theta \tag{S36}$$

Where $\omega(\vec{q})$ is the relaxation rate for each $\vec{q}$, mode, given by the expression:

$$\omega(q) = \frac{e^{(-2qd)}\left[-1 + e^{(2qd)} - 2(qd) - 2(qd)^2\right]}{4\eta q} \left(k_c q^4 + \sigma_{ef} q^2 + \gamma\right) \tag{S37}$$

In this equation, η represents the viscosity of the cytoplasm between the free and adhered membranes. Considering the curvature fluctuation model in the regime of a free

membrane, that is, $d \to \infty$ e $\sigma_{ef} = \gamma = 0$ the expression for the relaxation rate simplifies to:

$$\omega(q) = \frac{k_c q^3}{4\eta} \tag{S38}$$

With these approximations for the relaxation rate and the spatial fluctuation spectrum, the temporal autocorrelation function becomes:

$$= \frac{2(\Delta n k_0)^2}{(2\pi)^2} \int_0^{q_{max}} q d\mathrm{q} \int_0^{2\pi} \left(\frac{k_b T}{k_c q^4}\right) e^{\frac{-k_c\, q^3\, t}{4\eta}} \left(\frac{z_f^2\, q^4}{4k^2}\right) d\theta \tag{S39}$$

By calculating the angular part of the integral and reorganizing the expression, we have:

$$= 2\pi \frac{2(\Delta n k_0)^2}{4\pi^2} \frac{Z_f{}^2}{4k^2} \frac{k_b T}{k_c} \int_0^{q_{max}} q e^{\frac{-k_c\, q^3\, t}{4\eta}} dq \tag{S40}$$

$$= 2\pi \frac{2(\Delta n k_0)^2}{4\pi^2} \frac{Z_f{}^2}{4k^2} \frac{k_b T}{k_c} \int_0^{q_{max}} q e^{\frac{-k_c\, q^3\, t}{4\eta}} dq \tag{S41}$$

$$= \left(\frac{\Delta n k_0}{k}\right)^2 \frac{Z_f{}^2}{4\pi} \frac{k_b T}{k_c} \int_0^{q_{max}} q e^{\frac{-k_c\, q^3\, t}{4\eta}} d\mathrm{q} \tag{S42}$$

We know that $\left(\frac{\Delta n k_0}{k}\right)^2 = \left(\frac{\Delta n}{n_0}\right)^2$, and we finally obtain the expression that represents the model treated in this work as a tool for obtaining the biomechanical parameters k_c e η:

$$< \Delta C\,(0,0) \Delta C(0,t) > \; = \left(\frac{\Delta n}{n_0}\right)^2 \left(\frac{Z_f{}^2}{4\pi}\right) \left(\frac{k_b T}{k_c}\right) \int_0^{q_{max}} q e^{\frac{-k_c\, q^3\, t}{4\eta}} dq \tag{S43}$$

References

1. Mandel, L.; Wolf, E.; Meystre, P. Optical Coherence and Quantum Optics. *American Journal of Physics* 1996, 64(11), 1438–1439, https://doi.org/10.1119/1.18450.
2. Goodman. J.W. Fresnel diffraction. *In Introduction to Fourier Optics*; 4th ed. W.H. Freeman: New York, 2017; Chapter 4, pp. 57-85. ISBN 978-0-07-023776-6.
3. Roma, P.M.S.; Siman, L.; Amaral, F.T.; Agero, U.; Mesquita, O.N. Total Three-Dimensional Imaging of Phase Objects Using Defocusing Microscopy: Application to Red Blood Cells. *Applied Physics Letters* 2014, *104 (25)*, 251107, https://doi.org/10.1063/1.4884420.
4. Glionna, G.; Oliveira, C.K.; Siman, L.G.; Moyses, H.W.; Prado, D.M.U.; Monken, C.H.; Mesquita, O.N. Tomography of Fluctuating Biological Interfaces Using Defocusing Microscopy. *Applied Physics Letters* 2009, 94(19), 193701, https://doi.org/10.1063/1.3136842.

Disclaimer/Publisher's Note: The statements, opinions and data contained in all publications are solely those of the individual author(s) and contributor(s) and not of MDPI and/or the editor(s). MDPI and/or the editor(s) disclaim responsibility for any injury to people or property resulting from any ideas, methods, instructions or products referred to in the content.
